# Complete Genome Sequence of Vaccinia Virus Strain L-IVP

**DOI:** 10.1128/genomeA.00372-16

**Published:** 2016-05-12

**Authors:** Alexander N. Shvalov, Galina F. Sivolobova, Elena V. Kuligina, Galina V. Kochneva

**Affiliations:** a State Research Center of Virology and Biotechnology “Vector,” Koltsovo, Novosibirsk Region, Russia; b Institute of Chemical Biology and Fundamental Medicine SB RAS, Novosibirsk, Russia

## Abstract

Most of the live vaccine doses of vaccinia virus donated to the Intensified Smallpox Eradication Programme after 1971 were prepared using the L-IVP strain. A mixture of three clones of the L-IVP strain was sequenced using MySEQ. Consensus sequence similarity with the vaccinia virus Lister strain is 99.5%.

## GENOME ANNOUNCEMENT

Due to its low pathogenicity, good cultivation properties, and ease of use for genomic manipulations, vaccinia virus is very promising and safe to use as a recombinant vaccine, gene transfer vector, and for other applications (1). The L-IVP vaccinia virus strain was derived by adapting the Lister strain of vaccinia virus to calf skin at the Institute for Research on Virus Preparations, Moscow, former Soviet Union (2). The L-IVP strain was used for the production of most of the vaccines donated to the Intensified Smallpox Eradication Programme after 1971 (3). Both nonrecombinant and recombinant variants of the L-IVP strain demonstrated high levels of tumor cell selectivity and oncolytic activity (4–6).

The original wild-type vaccinia virus Lister strain (wt-Lister) as well as the L-IVP strain consist of numerous clones. Besides the currently presented sequence, there is only one recombinant strain derived from the vaccinia virus L-IVP strain that has been sequenced—GLV-1h68 (GenBank accession no. EU410304.1) (7). There are also complete genomes of 4 other variants of Lister strain clones, an averaged wt-Lister strain genome (GenBank accession no. AY678276.1), and 71 other complete sequences of vaccinia virus strains.

Strain L-IVP was obtained from the State Collection of Viral and Rickettsial Disease Agents of the State Research Center of Virology and Biotechnology “Vector.” A mixture of three clones, derived from equal size plaques induced by the vaccinia virus L-IVP strain on a monolayer of CV-1 cells, was passed 6 times in cells of the same type and purified by centrifugation in sucrose density gradient.

Strain L-IVP was sequenced on an Illumina Miseq platform using paired-end sequencing with 150-nt tag length and a mean insertion size of 385 nt. A consensus sequence was constructed using Mira v4.9.1 and Tmap v4.2.18. The length of the resulting sequence was 186,498 with an average coverage of 79×. The resulting sequence was annotated using GATU (8).

There were ~1,000 single nucleotide polymorphisms (SNPs) and ~70 indels due to variations between the clones. Six of these indels lead to shifts of open reading frames (ORFs). One deletion leads to fusion of A39R and 169 ORFs. This fusion also occurs in several other vaccinia strains. The most noticeable difference is the deletion in two of three clones of the region 9,670 to 13,414 and the insertion of sequence CTATTACACCGGCTGAGT between C9L and C10L.

Phylogenetic analysis using the maximum likelihood method with a bootstrap test of vaccinia strains showed clusterization of the L-IVP sequence within the Lister subfamily. There are no major changes in the genome structure of the L-IVP sequence and the nearest published sequence (wt-Lister). The overall difference between them is ~50 indels and ~530 mismatches (conserved genome sequence similarity is 99.5%.).

The genome of L-IVP-derived GLV-1h68 (excluding recombinant inserts), another strain closely related to the L-IVP strain, differs from our consensus sequence much more. Besides the ~700 SNPs and ~50 small indels, in the GLV-1h68 genome there are extra copies of GL277 to GL283 ORFs and GL274 to GL276 ORFs are absent in the L-IVP strain genome.

Sequencing was performed in the SB RAS Genomics Core Facility, Novosibirsk, Russia.

### Nucleotide sequence accession number.

This genome sequence has been deposited in GenBank under the accession number KP233807.
